# Phylogenetic evidence for inter-typic recombination in the emergence of human enterovirus 71 subgenotypes

**DOI:** 10.1186/1471-2180-6-74

**Published:** 2006-08-30

**Authors:** Chan Yoke-Fun, Sazaly AbuBakar

**Affiliations:** 1 Department of Medical Microbiology, Faculty of Medicine, Universiti Malaya, 50603 Kuala Lumpur, Malaysia

## Abstract

**Background:**

Human enterovirus 71 (EV-71) is a common causative agent of hand, foot and mouth disease (HFMD). In recent years, the virus has caused several outbreaks with high numbers of deaths and severe neurological complications. Several new EV-71 subgenotypes were identified from these outbreaks. The mechanisms that contributed to the emergence of these subgenotypes are unknown.

**Results:**

Six EV-71 isolates from an outbreak in Malaysia, in 1997, were sequenced completely. These isolates were identified as EV-71 subgenotypes, B3, B4 and C2. A phylogenetic tree that correlated well with the present enterovirus classification scheme was established using these full genome sequences and all other available full genome sequences of EV-71 and *human enterovirus A *(HEV-A). Using the 5' UTR, P2 and P3 genomic regions, however, isolates of EV-71 subgenotypes B3 and C4 segregated away from other EV-71 subgenotypes into a cluster together with coxsackievirus A16 (CV-A16/G10) and EV-71 subgenotype C2 clustered with CV-A8. Results from the similarity plot analyses supported the clustering of these isolates with other HEV-A. In contrast, at the same genomic regions, a CV-A16 isolate, Tainan5079, clustered with EV-71. This suggests that amongst EV-71 and CV-A16, only the structural genes were conserved. The 3' end of the virus genome varied and consisted of sequences highly similar to various HEV-A viruses. Numerous recombination crossover breakpoints were identified within the non-structural genes of some of these newer EV-71 subgenotypes.

**Conclusion:**

Phylogenetic evidence obtained from analyses of the full genome sequence supports the possible occurrence of inter-typic recombination involving EV-71 and various HEV-A, including CV-A16, the most common causal agent of HFMD. It is suggested that these recombination events played important roles in the emergence of the various EV-71 subgenotypes.

## Background

More than sixty different types of human enteroviruses have been identified [1]. Species *human enterovirus A *(HEV-A) which include 11 members of the coxsackievirus A (CV-A) group; CV-A2, CV-A3, CV-A4, CV-A5, CV-A6, CV-A7, CV-A8, CV-A10, CV-12, CV-14, CV-A16 and human enterovirus 71 (EV-71) are associated with several human diseases [1,2]. CV-A2, CV-A6, CV-A8 and CV-A10 are known to cause herpangina [2]; CV-A2, CV-A4, CV-A7 and CV-A10 cause aseptic meningitis [2] and CV-A5, CV-A10, CV-A16 and EV-71 usually cause hand, foot and mouth disease (HFMD) [2]. Amongst these HEV-A viruses, only EV-71 is associated with major outbreaks involving infections with severe neurological complications and deaths. Recent outbreaks in Malaysia, Taiwan and Vietnam claimed more than 130 deaths [3,4]. In these outbreaks, EV-71 was isolated from patients' specimens and post-mortem tissues, suggesting its direct involvement as the etiologic agent [5,6]. Molecular epidemiological investigations performed using the partial gene sequences of all available EV-71 isolates suggested the presence of several EV-71 lineages co-circulating during most of the Asian outbreaks [3,6-11]. EV-71 subgenotypes B3, B4, C1 and C2 were reported in Malaysia with subgenotype B3 being the most prevalent in the 1997 outbreak [3,7-9]. The subgenotype B4 was present initially at a low level but became endemic and eventually replaced subgenotype B3 as the predominant virus in the subsequent outbreaks in Malaysia and the region [7,9]. Neither the B3 nor B4 subgenotype, however, was associated with any fatal infections outside the Malaysia 1997 outbreak. Similarly, the C2 isolates were identified as the main causal agent of the HFMD-associated severe and fatal infections during the 1998 Taiwan outbreak [6,10]. These isolates also caused severe infections in the Perth 1999 outbreak [7,9] but no report of serious infection or death attributed to these isolates was recorded from the Malaysia 1997 outbreak [8,12]. Recently, two new EV-71 subgenotypes, C3 and C4 were proposed to accommodate EV-71 strains isolated from cases of aseptic meningitis and paralysis in Korea and China, respectively [7,13,14]. There were no reports, however, that the subgenotype C4 isolates were associated with severe infections [13,14]. The emergence of the various EV-71 subgenotypes with varying degree of clinical severity is puzzling. The viruses together with other HEV-A viruses are known to be present in the region and usually cause the typical mild HFMD [7-9,15]. In the present study, the potential mechanisms that could play important role in the emergence of the different EV-71 subgenotypes are phylogenetically investigated.

## Results

### Phylogenetic relationships between EV-71 and other HEV-A viruses

The complete genome sequences of all the EV-71 isolates were aligned with other available HEV-A complete genome sequences obtained from the Genbank. The total length of the genomes varied between 7395–7434 nucleotides and they encode for 2189–2201 amino acids. The differences in genome length were caused by the presence of deletions or insertions within the 5' UTR, capsid gene and 3' UTR sequences. The capsid genome region of EV-71 was longer (1 – 4 amino acids) or of the same length with other HEV-A viruses, with the exception of CV-A4 and CV-A6 which have eight extra amino acid insertions. No deletions and insertions were observed in the P2 and P3 genomic regions, hence, the gene length within these genomic regions remained constant for all the HEV-A viruses.

**Figure 1:**
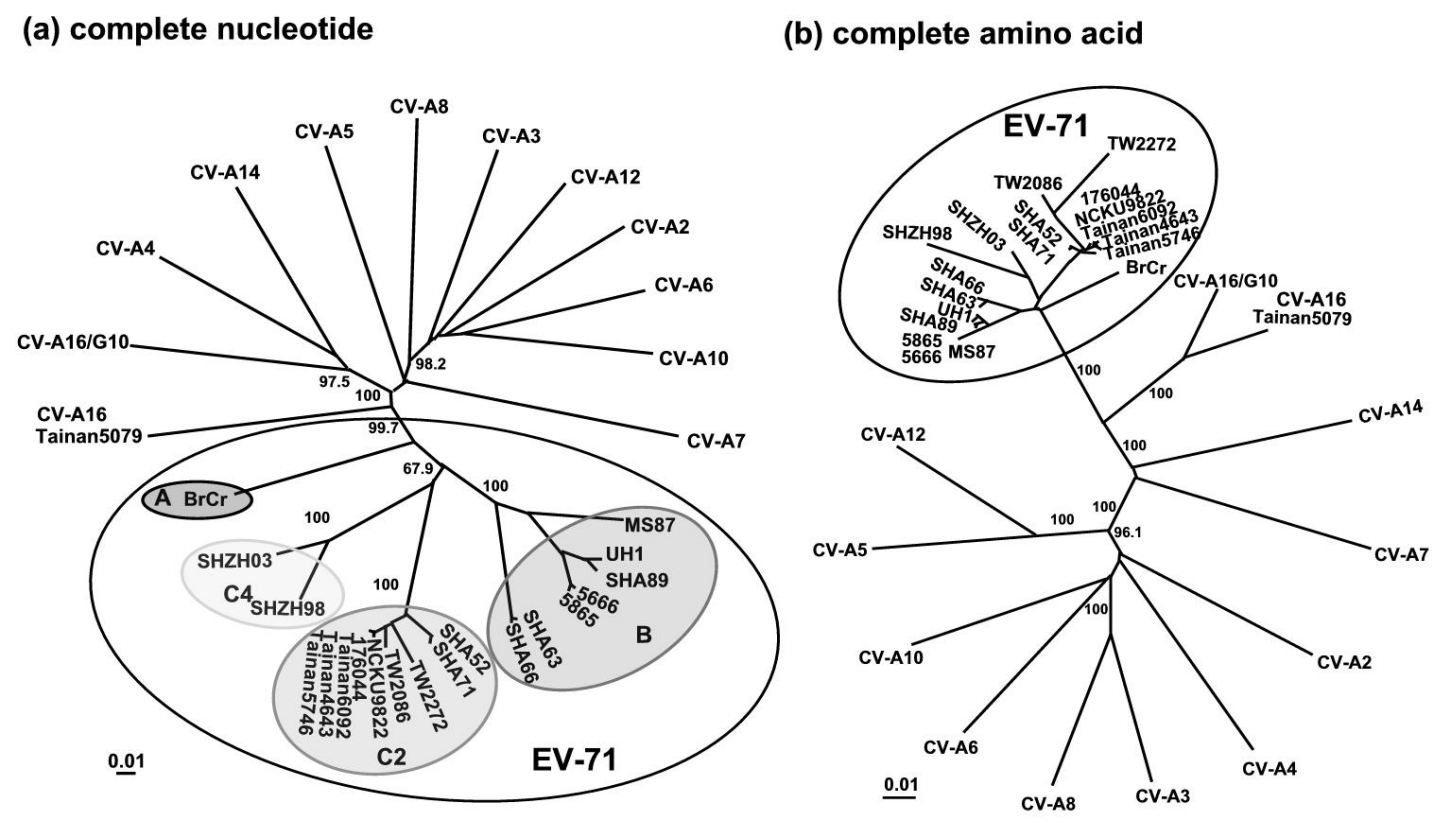
Phylogenetic relationships amongst HEV-A isolates. The neighbour-joining tree was established from alignments of the full genome nucleotide (a) and amino acid sequences (b). The percentage of bootstrap replicates supporting the trees are indicated at the nodes. The branch lengths are proportional to the genetic distances corrected using Kimura-two-parameter substitution model.

EV-71 VP1 gene sequences had an average sequence divergence of 17–22% at nucleotide level and 3–8% at amino acid level, respectively. The average sequence divergence between EV-71 VP1 and other HEV-A viruses was 22–29% at the nucleotide level and 10–20% at amino acid level, respectively (not shown). These suggested that EV-71 in general, were distinct from all the other existing HEV-A viruses and using the EV-71 VP1 gene sequence, several subgenotypes were identified [7,9,16,17]. An unrooted phylogenetic tree constructed using the whole genome sequences of all EV-71 and HEV-A viruses had all the EV-71 isolates subdivided into three genotypes, A, B (B2, B3 and B4) and C (C2 and C4). The phylogenetic tree was similar to that constructed using the VP1 gene sequence with all the HEV-A viruses clustered into their respective groups (Figure 1a). A similar tree topology depicting the characteristic grouping of EV-71 and other HEV-A viruses was obtained using the complete amino acid sequences (Figure 1b).

**Figure 2:**
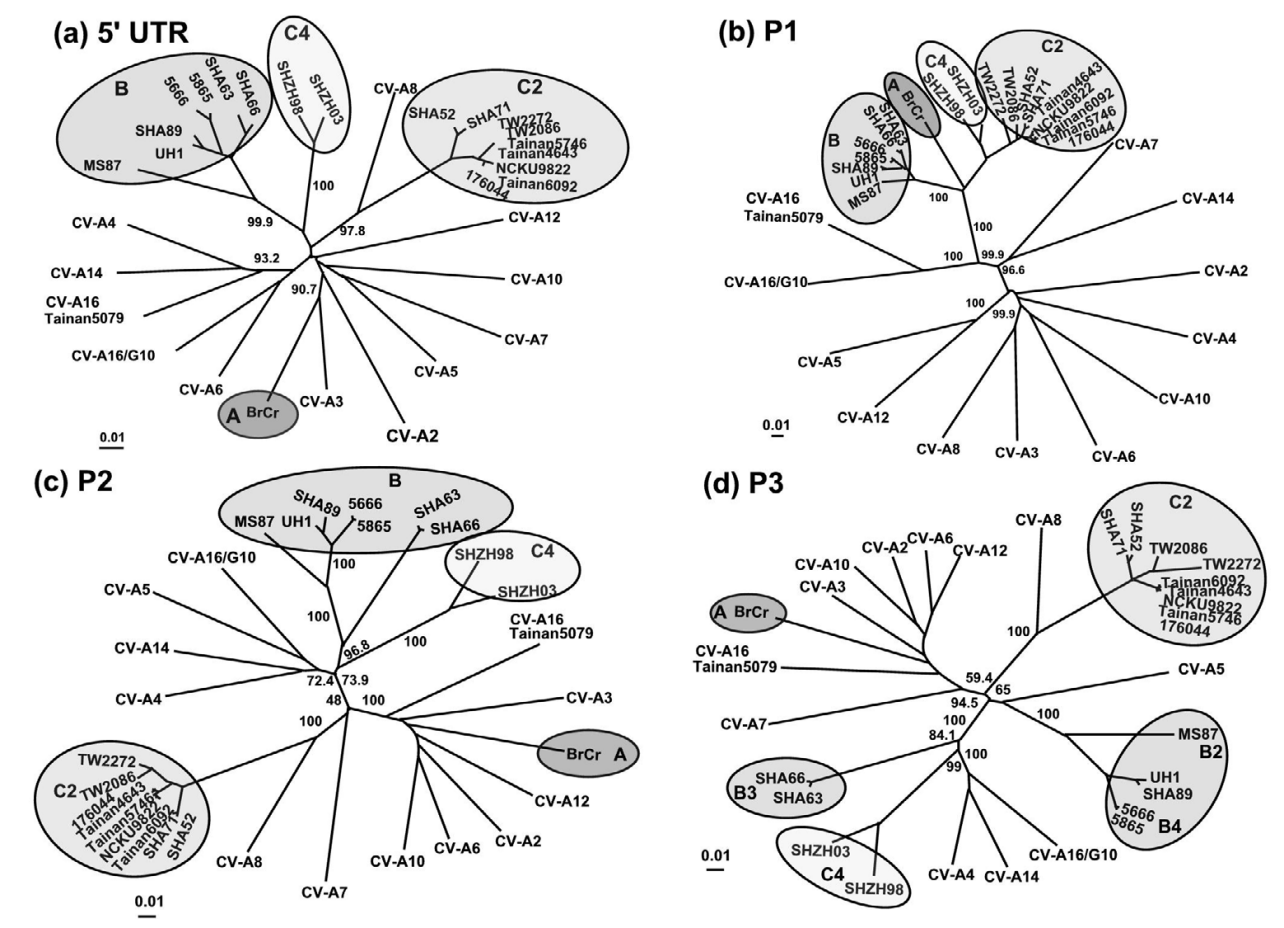
Unrooted phylogenetic trees showing the relationships amongst HEV-A isolates using the different genomic regions. The neighbour-joining trees were constructed from alignment of the 5' UTR (a), P1 (b), P2 (c) and P3 genomic region (d), respectively. The percentage of bootstrap replicates supporting the trees are indicated at the nodes. The branch lengths are proportional to the genetic distances corrected using Kimura-two-parameter substitution model.

At the 5' UTR genomic region, the prototype EV-71, BrCr branched out together with CV-A2 and CV-A3, and segregated away from the rest of the EV-71 genotypes. Isolates of EV-71 subgenotype C2 were phylogenetically closer to CV-A8 and this segregation was supported by a high bootstrap value (Figure 2a). EV-71 genotype B and C4 remained as one cluster, while CV-A4, CV-A14 and two CV-A16, CV-A16/G10 and Tainan5079 grouped into another distinct cluster. At the P1 genomic region, no segregation of any EV-71 isolates was observed and all the other HEV-A isolates were distantly related to one another (Figure 2b). The tree topology was very similar to that drawn using complete amino acid sequences (not shown). Based on the tree drawn using the P1 genomic region, at least six clustering patterns were observed; (i) all the EV-71 isolates together with CV-A16, (ii) CV-A2 and CV-A4, (iii) CV-A3 and CV-A8, (iv) CV-A5 and CV-A12, (v) CV-A6 and CV-A10 and (vi) CV-A7 and CV-A14. These patterns received high bootstrap support (> 95%) and corresponded to the neutralization cross-reactivity patterns observed previously [18].

At the P2 genomic region, EV-71 BrCr clustered with CV-A2, CV-A3, CV-A6, CV-A10 and CV-A12, while the EV-71 subgenotype C2 isolates were phylogenetically closer to CV-A8 (Figure 2c). EV-71 isolates of subgenotype B2 and C4 segregated from all other EV-71 and formed a cluster together with CV-A4, CV-A5, CV-A14 and CV-A16/G10 (Figure 2c). CV-A16 Tainan5079 segregated away from the prototype CV-A16/G10 and grouped with EV-71 BrCr, CV-A2, CV-A3, CV-A6, CV-A10 and CV-A12 (100% bootstrap support, Figure 2c). At the P3 genomic region, the different EV-71 lineages formed four distinct clusters together with other HEV-A viruses. Isolates of subgenotype C2 were phylogenetically closer to CV-A8 and this was supported by a high bootstrap value (100%). Isolates of subgenotype B3 and C4 isolates clustered with CV-A4, CV-A14 and CV-A16 and support for the clustering was also significant (100% bootstrap). EV-71 isolates of subgenotype B2 and B4 were phylogenetically closer to CV-A5, although this did not receive high bootstrap support (65%). The genotype A isolate BrCr segregated from all the recent EV-71 subgenotypes and clustered with CV-A2, CV-A3, CV-A6, CV-A10 and CV-A12 (Figure 2d). At the P3 genomic region, isolate CV-A16 Tainan5079 segregated away from CV-A16/G10 and grouped with EV-71 BrCr, CV-A2, CV-A3, CV-A6, CV-A10 and CV-A12 (Figure 2d).

An unrooted phylogenetic tree drawn using only the 3' UTR showed clustering of EV-71 isolates of subgenotype of B3 and C4 with CV-A4, CV-A14 and CV-A16 (95% bootstrap support, not shown). No significant segregation (< 30% bootstrap support), however, was observed for the remaining isolates and this was perhaps due to the short sequence length of the 3' UTR. The clustering of isolates of subgenotype B3 and C4 with CV-A4, CV-A14 and CV-A16 at the 3' UTR genomic region was consistent with the previous clustering at the P2 and P3 genomic regions. Based on these results, it appeared that sequences of genes at the 3' half of the EV-71 genome contributed to the multiple and diverse EV-71 subgenotypes and these genes showed high similarity to different HEV-A viruses.

### Inter-typic recombination between EV-71 and other HEV-A viruses

**Figure 3:**
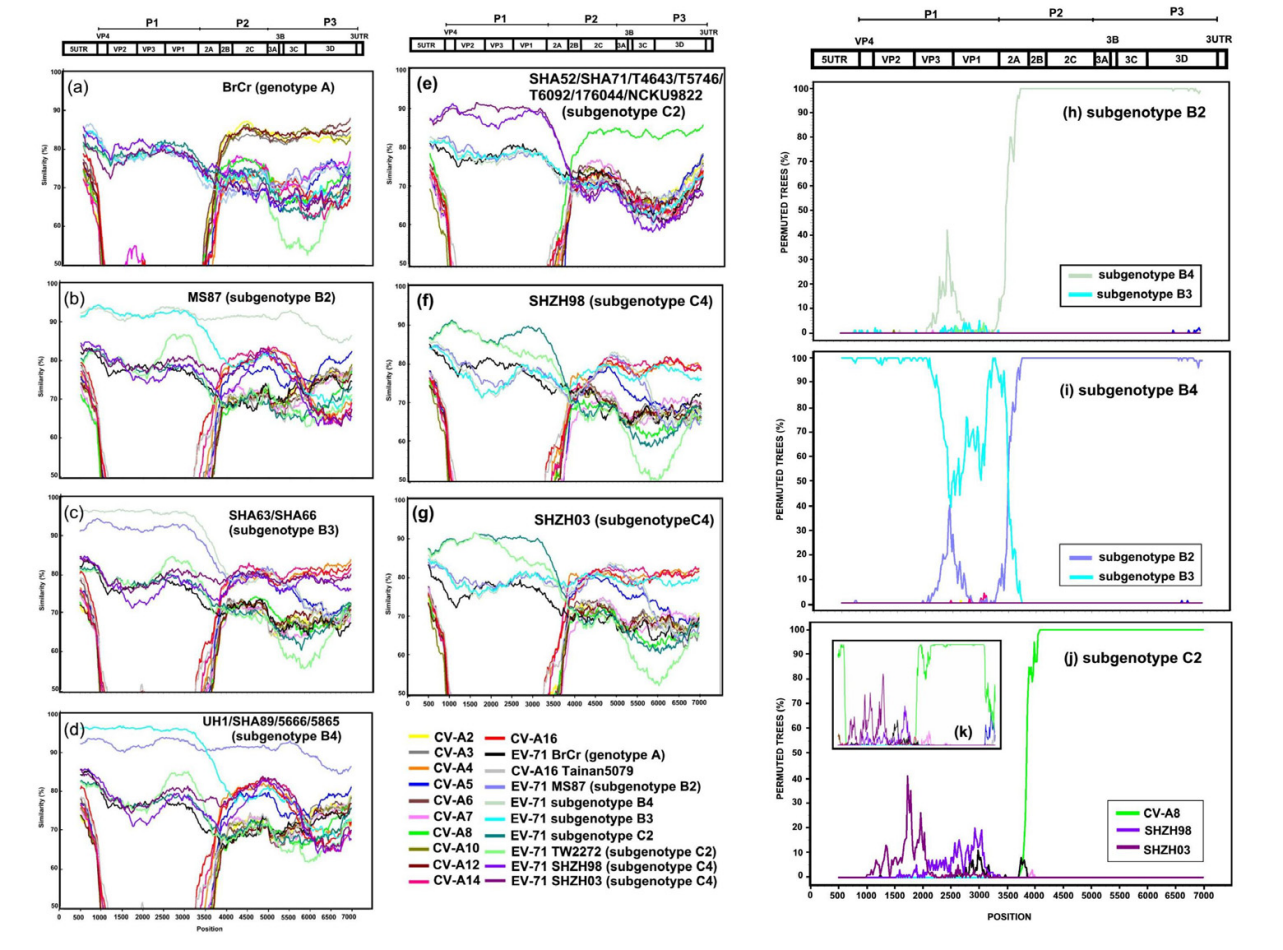
Emergence of EV-71 subgenotypes by recombination with other HEV-A viruses. Similarity plot analyses were performed using the full genome sequence of all EV-71 genotypes and subgenotypes. The sequences were queried against all other available HEV-A viruses. The nucleotide sequence of BrCr, genotype A (a), nucleotide sequence of MS87, subgenotype B2 (b), consensus nucleotide sequence of subgenotype B3 (c), consensus nucleotide sequence of subgenotype B4 (d), consensus nucleotide sequence of subgenotype C2 (e) SHZH98, subgenotype C4 (f) and SHZH03, subgenotype C4 (g) were used in the similarity plot analyses. Results from the bootscan analyses indicate the likelihood of clustering of subgenotype B2 isolate, MS87 (h), subgenotype B4 (i) and subgenotype C2 (j) isolates with their respective potential parental isolates. A sliding window of 1000 nucleotides was moved in increments of 20 nucleotides at a time. All gaps were stripped and the sequences were corrected for multiple substitutions. A transition to tranversion ratio of ten was used and 50% consensus files were used to exclude the poorly conserved sites. The subgenotype C2 isolates (k) were also queried against other HEV-A viruses using a window size of 400 nucleotides in increments of 20 nucleotides per slide. Bootstrap values of > 70% were used to indicate robust support for the tree topology [43]. The different HEV-A viruses and EV-71 genotypes are represented by the different line colors.

The conflicting tree topologies in the 5' UTR, P2 and P3 genomic regions raised the possibility that the different EV-71 genotypes contained chimeric genome sequences, perhaps as a result of a previous recombination event. Similarity plot analysis performed using consensus sequences of each EV-71 subgenotype against other EV-71 subgenotypes and HEV-A viruses showed that the P1 genomic region of all the EV-71 genotypes have relatively high genome sequence similar to each other (81–82%) with very low sequence similarity (< 50%) to other HEV-A viruses. In the P2 and P3 genomic regions, however, three different similarity patterns for all the EV-71 isolates were observed. The first pattern grouped EV-71 genotype A, BrCr with CV-A2, CV-A3, CV-A6, CV-A10 and CV-A12. These viruses shared relatively high sequence similarity (83–86%) to each other, and with low sequence similarity to other EV-71 genotypes (Figure 3a). The second pattern grouped EV-71 isolates to their respective genotypes. Isolates of genotype B were highly similar to each other for examples, subgenotype B2, MS87 had high sequence similarity to subgenotype B4 (≥ 88%, 100% bootstrap support, Figure 3b, 3h) and vice versa, subgenotype B4 isolates had high sequence similarity to the subgenotype B2 (≥ 91%, 100% bootstrap support, Figure 3d, 3i). The third pattern included EV-71 isolates of subgenotype B3 and C4. These isolates had high sequence similarity to EV-71 genotype B, CV-A4, CV-A14 and CV-A16/G10 at P2 genomic region (≥ 81%) and high sequence similarity to CV-A4, CV-A14 and CV-A16/G10 at P3 genomic region ((≥ 83%), Figure 3c, 3f, 3g). At the P3 genomic region, the sequence similarity of isolates of subgenotype B3 and C4 to the rest of the EV-71 genotypes was only between 75–79%. Similarly, EV-71 of subgenotype C2 shared higher sequence similarity to CV-A8 (≥ 84%) and low sequence similarity to other existing EV-71 genotypes (≤ 78%, Figure 3e). These phylogenetic segregations received a high bootstrap support value (Figure 3j). Using a smaller scanning window size of 400 nucleotides, the 5' UTR of subgenotype C2 isolates also showed high sequence similarity to CV-A8 (Figure 3k).

**Figure 4:**
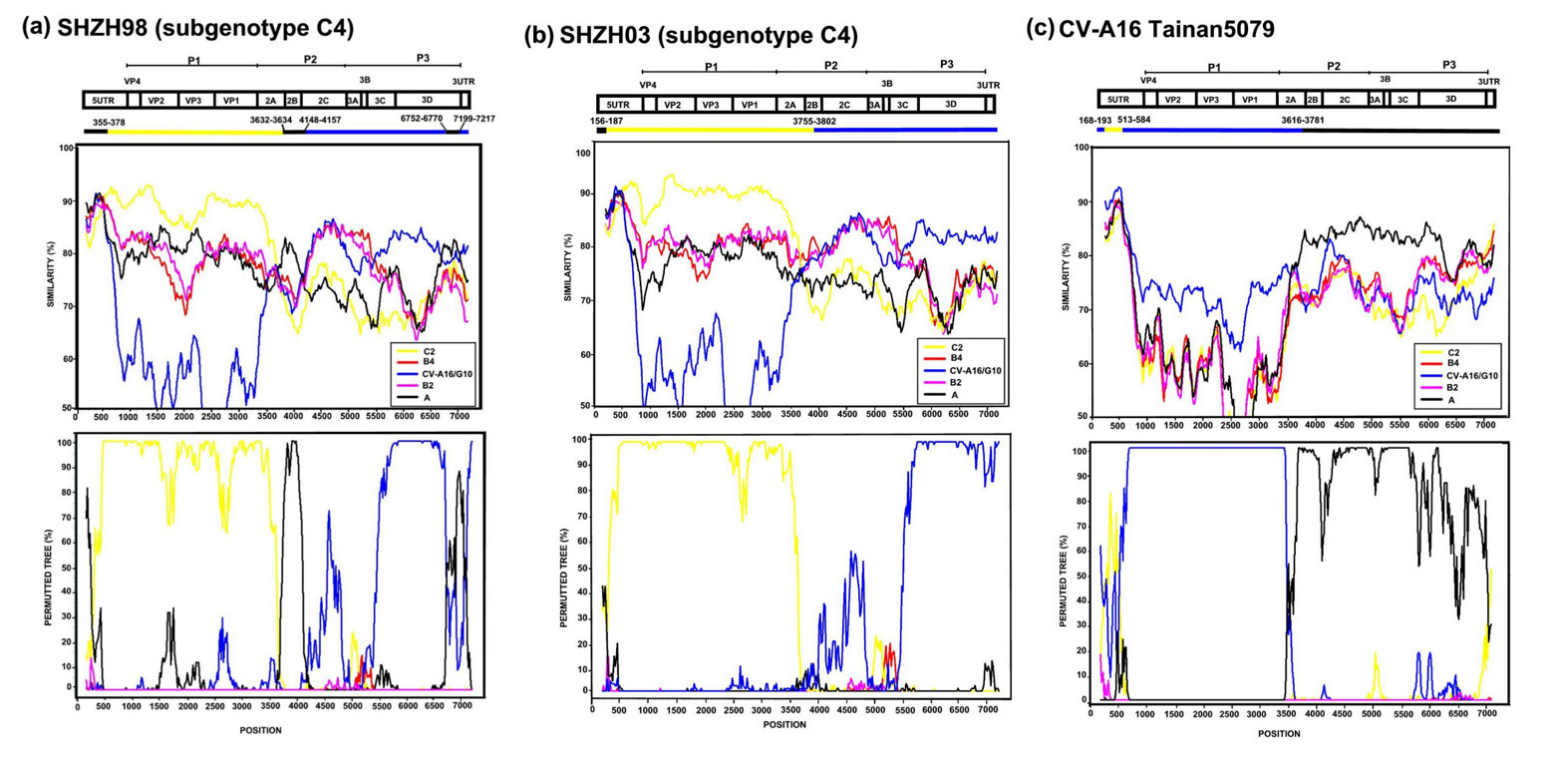
Identification of recombinant sequences in the genome of EV-71 subgenotype C4 and CV-A16 Tainan 5079. The organization of the EV-71 genome, the respective crossover breakpoints and potential parental sequences are shown. The upper panel shows results of a pairwise comparison between SHZH98 (a), SHZH03 (b) and CV-A16 Tainan 5079 (c) genome sequences against the consensus sequences of EV-71 genotypes A, B2, B4, C2, and CV-A16/G10. The lower panel shows results from the bootscan analysis which illustrates the likelihood of clustering of the putative recombinants with respect to the parental virus. A window size of 400 nucleotides in increments of 20 nucleotides at a time was used. Positions containing gaps were excluded from the comparison.

Evidence of multiple recombination crossover points with at least five breakpoints delineating the whole genome was obtained from the similarity plot analysis of EV-71 subgenotype C4 isolate, SHZH98 (Figure 4a). For this analysis, a smaller scanning window size of 400 nucleotides and sequences of EV-71 isolates were used together with CV-A16/G10 as the outgroup virus. This was done to overcome the phylogeny noise problem. Three genomic regions nucleotides 1–355, 3634–4148 and 6770–7199 showed 88%, 82% and 84% genome sequence similarity to EV-71 genotype A, respectively. One genomic region (nucleotides 378–3632) shared ≥ 88% sequence similarity to EV-71 subgenotype C2 while another genomic region (nucleotides 4157–6752) had 83% sequence similarity to CV-A16/G10 (Figure 4a). The χ^2 ^values supporting the presence of EV-71 genotype C and CV-A16/G10 genome sequences within the genome of isolate SHZH98 were maximal at the genomic region spanning nucleotides 378–3632 and 4157–6752, respectively. These results reflect possible mosaicism of isolate SHZH98 genome involving sequences of EV-71 genotype C structural genes, CV-A16/G10 non-structural genes and the remnants or perhaps fossilized ancestral sequences of the prototype EV-71 BrCr, interspersed within the genome. It was noted, however, that the BrCr-like sequences were no longer found in the non-structural regions of the more recent subgenotype C4 virus isolate, SHZH03 (Figure 4b) and based on the maximum χ^2 ^values, only two crossover points were located within nucleotides 156–187 (5' UTR) and 3755–3802 (2A/2B junction). At the 5' UTR, however, the first 150 nucleotides were still similar to BrCr sequences but the latter crossover point switched the genome from subgenotype C2 to CV-A16-like sequences with no insertion of BrCr sequences. These results provided additional evidence that EV-71 subgenotype C4 isolates may have emerged due to recombination between subgenotype C2 and CV-A16/G10-like viruses.

Support for inter-typic recombination involving CV-A16 and EV-71 were also obtained from the phylogenetic investigation of the CV-A16 Tainan5079. Several recombination breakpoints were revealed when the CV-A16 Tainan5079 whole genome sequence was queried against several potential parental genome sequences (Figure 4c). A major recombination breakpoint was identified between nucleotides 3616–3781 within the 2A gene (≥ 84% sequence similarity), hence switching the CV-A16 Tainan5079 genome sequences to that of the non-structural gene sequences of EV-71 BrCr (nucleotides 3781–7409). Two additional breakpoints were noted between nucleotides 168–193 and 513–584 of the 5' UTR sequences switching the genome sequence from CV-A16-like to that of EV-71 subgenotype C2 (Figure 4c). Acquisition of the EV-71 subgenotype C2 sequences also resulted in the CV-A16 Tainan5079 isolate acquiring the EV-71-like 5' UTR RNA stem loop structure (not shown). When the amino acid sequences of the non-structural genes were examined, at least 45 amino acids were found to be similar to that encoded by the ancestral EV-71 BrCr and not to CV-A16 G/10. Of these amino acids, 21 amino acid differences occurred within the 3D RNA polymerase gene alone, suggesting that the gene was very much EV-71-like than CV-A16 (not shown). These results revealed that the recently isolated CV-A16 had most of its genome 5' half including all the structural genes except the 5' UTR consisting of CV-A16-like sequences and the remaining 3' half of the genome consisting of the supposedly no longer circulating EV-71 genotype A BrCr sequence.

## Discussion

Results from the full genome sequence similarity plot analyses and phylogenetic investigations presented here support the possible occurrence of inter-typic recombination involving EV-71 and various HEV-A, including CV-A16, the common causal agent of HFMD. All the EV-71 isolates examined had conserved 5' half of the genome, which consisted of mainly the structural genes including VP1, VP2, VP3 and VP4. These structural gene sequences particularly VP1 and VP4 when used to construct the phylogenetic trees clustered all EV-71 isolates into a unique clade of its own, away from other HEV-A viruses. This justifies usage of these genes for the molecular identification of EV isolates [19,20]. It is possible that the structural genes especially the virus capsid gene, carries genetic restrictions required to ensure virus receptor recognition, binding and entry into host cells, and hence remain highly conserved. CV-A16-like sequences were present within the genome 3' end of EV-71 subgenotype B3 and C4. Conversely, EV-71-like sequences were also found within the genome 3' end of CV-A16 Tainan5079. These highlight the possible occurrence of recombination events in the wild between the two common causal agents of HFMD. This possibility is not difficult to envisage considering that both EV-71 and CV-A16 viruses are known to co-circulate within the same population and cause a common disease. It is particularly true in parts of the world where the level of hygiene practices and sanitation are lacking or less developed, as in these circumstances the possibility of co-infection is expected to be higher.

Results from the present study also suggest that inter-typic recombination events occurred between EV-71, CV-A16 and other HEV-A viruses. The incongruent phylogeny patterns observed suggest the possible occurrence of recombination in EV-71 in a similar manner to those previously reported for a number of enteroviruses [21-27]. It is reported that most of the current circulating HEV-B viruses are actually recombinants relative to its prototype strains isolated in the 1950s [23,27]. Recombination amongst HEV-A viruses has also been described in CV-A6, CV-A10 and CV-A12 [18] and a novel group of HEV-A consisting of EV-76, EV-89, EV-90 and EV-91 [28]. The P1 genomic region of EV-70, a virus that causes acute haemorrhagic conjunctivitis is highly similar to EV and rhinovirus. In addition, the P2 and P3 genomic regions are phylogenetically closer to CV-B and swine vesicular disease virus [29]. Another EV, CV-B5, has its major capsid proteins that are most closely related to swine vesicular disease virus, with the 5' and 3' UTR and the P3 genomic region that are highly similar to the corresponding regions of other CV-B viruses [30]. Other example of EV with highly diverse genome sequence origin includes the prototype EV-73 isolated in California in 1988. This EV has P1 genomic region that is highly similar to echovirus 6, echovirus 29, echovirus 21 and echovirus 30, with the P2 and P3 genomic regions that share high similarity to CV-B3 [31]. Together, these findings reiterate the suggestion that recombination is one of the potential mechanisms by which genetic diversity, is generated in EV-71 and other HEV-A viruses especially within the non-structural genes where recombination hot spots are mainly located [21,23,32-35]. While recombination could occur within the structural gene region, it perhaps yielded nonviable or unstable progeny viruses that did not survive and this could be due to generation of less fit variants with deleterious mutations [36]. Selection of a better fit virus is likely to have contributed towards the demise of the recombinant EV-71 B3 virus once found prevalent in Peninsula Malaysia and Sarawak during the 1997 outbreak, Singapore and Japan in 1997 [7,8] and Perth in 1999 [9]. This virus was shown to replicate less efficiently in comparison to EV-71 B4 viruses [37], hence it could be the reason why it was rapidly replaced with EV-71 B4 viruses which remained endemic in the region for at least up to 2002, causing infections in Sarawak, Singapore, Taiwan and Japan [7,8]. The acquisition of a segment of genes from CV-A16 by EV-71 B3 virus [21,37] could have rendered the virus less fit to adapt to a new environment especially the host immunity.

## Conclusion

Phylogenetic evidence of possible inter-typic recombination amongst HEV-A viruses including between the two causal agents of HFMD, EV-71 and CV-A16, were obtained from analyses using the virus isolates' full genome sequences. The finding highlights the potential role of recombination in the emergence of the newer EV-71 lineages including those perhaps with varied virulence and clinical manifestations. Active molecular surveillance of EV-71 and CV-A16 would help to ensure rapid identification of emergence of any potentially highly virulent EV-71 or CV-A16 strains. This is particularly pertinent in regions where recurring HFMD outbreaks involving the different HEV-A have occurred.

## Methods

### RNA isolation and sequencing

Six EV-71 isolates initially identified into the different genotypes using the 5' UTR sequences were completely sequenced (Table 1). Purified total RNA of EV-71-infected Vero cells at either passage two or three were extracted using the TRI Reagent^® ^(Molecular Research Centre, Inc., USA) following the manufacturer's recommended protocols. Prior to the amplification, virus genomic RNA was reverse transcribed to generate cDNA fragments using oligo dT primers (Promega, USA) and EV-71 specific reverse primers. A total of eight sets of specific primers designed based on the published EV-71 sequences were used to obtain the complete genome sequences (Table 2). The PCR parameters for all the primer pairs were: 95°C for 3 min, followed by 30 cycles of 95°C for 1 min, 54°C for 1 min and 72°C for 1 min and final extension of 5 min at 72°C except annealing at 50°C for EV8 primer pair. The amplified DNA fragments were purified using QIAquick Miniprep kit (Qiagen, Germany) and sequenced directly. In instances when direct sequencing results were poor, the amplified DNA fragments were ligated into pGEM^®^-T cloning vector (Promega, USA) for transformation into Top 10F' *Escherichia coli *(Invitrogen, USA). DNA inserts were sequenced in both directions using the T7 and SP6 primers with ABI PRISM BigDye^® ^Terminator kit and ABI Prism 377 DNA sequencer (Applied Biosystems, USA).

### Sequencing analysis

Overlapping DNA sequences with at least 85% sequence homology and a minimum of 20 overlaps were assembled into contigs to generate consensus sequences using Sequencher version 4.0.5 (Gene Codes Corporation, USA). The consensus sequences were aligned against other EV-71 and HEV-A complete genome sequences retrieved from the GenBank (Table 1) using Clustal X version 1.81 [38]. All completely sequenced EV-71 isolates available in the GenBank and the six isolates obtained from the present study were used for construction of phylogenetic trees. The genetic distance was determined by a pairwise estimation of the sequences percent divergence. Positions with gaps were included, transition and transversion ratio was fixed at 10 and corrections for multiple substitutions were made. These options were chosen to take into account the ambiguous part of the alignments and to correct for more than one substitutions happening at many sites that may underestimate the actual genetic distances [38]. Phylogenetic trees presented here were constructed using the neighbour-joining method [39]. All the unrooted trees were displayed using TreeView version 1.66 [40]. The strength of the phylogenetic trees was estimated by bootstrap analyses using 1000 random samplings. A bootstrap value of ≥ 70% indicates a strong support for the tree topology [41]. Amino acid sequences were examined after stripping the 5' UTR and 3' UTR sequences and consensus sequences for each EV-71 genotype were established.

### Recombination analysis

Potential recombination amongst the isolates was determined as previously described [42]. Initially, the neighbour-joining trees were constructed using the various gene regions of the complete genome. The trees were manually inspected to determine if there was any shifting of the tree topology in comparison to that constructed using the whole genome sequence. The putative recombinant isolate, determined from the shifting of the tree topology, was then queried against all the existing HEV-A virus genomes. Initially, all the 31 manually corrected and aligned complete nucleotide sequences were used. Isolates that shared similar patterns were grouped together to generate consensus sequences. Recombination was identified when conflicting genome sequence profiles appeared suggesting acquisition of sequences from a different parental genotype. Phylogenetic trees were constructed for each of the putative recombinant sequences using the neighbour-joining tree algorithm and maximum likelihood distance model and support for the tree topology was determined by bootscanning analyses that utilized the bootstrapping procedures with 100 resamplings [42,43]. Bootstrap values of > 70% were used to indicate robust support for the tree topology [43]. The crossover breakpoints were identified when χ^2 ^values were at the maximum [44].

For the analysis of EV-71 and HEV-A viruses, all gaps were stripped and the sequences were corrected for multiple substitutions, transition to tranversion ratio of ten was used and 50% consensus files were used to exclude the poorly conserved sites. A sliding window of 1000 nucleotides was moved in increments of 20 nucleotides at a time and the resulting similarity values were plotted along the virus genome. Transition and transversion ratio of 10 was chosen based on that used for HEV-A viruses to avoid underestimation of actual genetic distances [18]. Both the similarity plot and bootscanning analysis sliding windows were based on that used for HEV-B to increase the phylogenetic signals and reduce the noise of unrelated phylogeny [33]. However, for the analysis amongst EV-71, the sequences were not corrected for multiple substitutions, and a transition to tranversion ratio of two and a sliding window of 400 nucleotides was used to map the possible recombination breakpoints [21].

## Abbreviation

CV Coxsackievirus

EV Enterovirus

HEV *Human enterovirus*

HFMD Hand, foot and mouth disease

UTR Untranslated region

## Competing interests

The author(s) declare that they have no competing interests.

## Authors' contributions

The corresponding author, Sazaly AbuBakar is the principal investigator of the study; was involved in the design, supervision, data analyses and writing of the report. Chan Y-F performed all the virological investigations, nucleotide sequencing and analyses of data. All authors were involved in the preparation of this "*Research Article*" and figures.

## Figures and Tables

**Table 1:** EV-71 and HEV-A sequences used for sequence analysis and construction of phylogenetic trees.

**Abbreviation**	**Strain**	**Accession number**	**Clinical presentation**	**Reference**
UH1 *	UH1/PM/1997	EMBL: AM396587	Fatal	3
SHA89 *	EV71/9/97/SHA89	EMBL: AJ586873	Fatal	21
SHA63 *	EV71/SAR/SHA63	EMBL: AM396588	HFMD	3
SHA66 *	EV71/SAR/SHA66	EMBL: AM396586	HFMD	3
SHA52 *	ENT/PM/SHA52	EMBL: AM396584	HFMD	11
SHA71 *	ENT/PM/SHA71	EMBL: AM396585	HFMD	11
5666	5666/sin/002209	EMBL: AF352027	HFMD	45
5865	5865/sin/000009	EMBL: AF316321	Fatal	45
BrCr	BrCr	Genbank: U22521	Aseptic meningitis	46
MS	MS/7423/87	Genbank: U22522	Aseptic meningitis	46
TW2272	TW/2272/98	EMBL: AF119795	Fatal	47
TW2086	TW/2086/98	EMBL: AF119796	HFMD	47
NCKU9822	NCKU9822	EMBL: AF136379	Fatal	6
176044	1245a/98/tw	EMBL: AF176044	HFMD	48
Tainan5746	Tainan/5746/98	EMBL: AF304457	HFMD	49
Tainan4643	Tainan/4643/98	EMBL: AF304458	Aseptic meningitis	49
Tainan6092	Tainan/6092/98	EMBL: AF304459	HFMD	49
SHZH98	SHZH98	EMBL: AF302996	HFMD	50
SHZH03	SHZH03	EMBL: AY465356	?	51
CV-A2	CV-A2/Fleetwood	EMBL: AY421760	Paralytic illness ^+^	18
CV-A3	CV-A3/Olson	EMBL: AY421761	Meningitis ^+^	18
CV-A4	CV-A4/High Point	EMBL: AY421762	Not known ^+^	18
CV-A5	CV-A5/Swartz	EMBL: AY421763	Poliomyelitis ^+^	18
CV-A6	CV-A6/Gdula	EMBL: AY421764	Meningitis ^+^	18
CV-A7	CV-A7/Parker	EMBL: AY421765	Bulbar poliomyelitis ^+^	18
CV-A8	CV-A8/Donovan	EMBL: AY421766	Sore throat, nuchal rigidity, muscle weakness ^+^	18
CV-A10	CV-A10/Kowalik	EMBL: AY421767	Meningitis ^+^	18
CV-A12	CV-A12/Texas-12	EMBL: AY421768	Flies in polio community ^+^	18
CV-A14	CV-A14/G-14	EMBL: AY421769	None ^+^	18
CV-A16/G10	CV-A16/G-10	Genbank: U05876	HFMD	52
Tainan5079	Tainan/5079/98	EMBL: AF177911	HFMD	49

* EV-71 isolates sequenced in the present study

^+ ^Reference: 2

**Table 2:** Oligonucleotide primers for cDNA synthesis and PCR.

**Primer set**	**Primer**	**Nucleotide sequence (5' to 3')**	Nucleotide position *	**Polarity**
EV1	EV1F	TAA AAC AGC YKK KGG KTK KYA CC	2–23	sense
	EV1R	TGM ACR TGR ATG CAR AAC C	1268–1286	antisense
				
EV2	EV2F	CCD GAT GTR YTR ACW GAA ACC	1200–1221	sense
	EV2R	ATC ACA TCT GCC ACY CTR TCY	2443–2463	antisense
				
EV3	EV3F	GGR GCR CCY AAY ACW GCY TAY ATW WTR GC	2333–2361	sense
	EV3R	CYT GRG CRG TRG TWG AHG AHA CKA RB	3451–3476	antisense
				
EV4	EV4F	CAR GTY TCH GTB CCR TTY ATG TCA CC	3005–3030	sense
	EV4R	GCT GTY TTB GMY TTR AYC CAV GC	4022–4044	antisense
				
EV5	EV5F	YAA CTC WMD SAG AAA RCA CTA YCC	3526–3559	sense
	EV5R	GWS GAK GCA ATS ACA AAC TTD GAK G	4719–4743	antisense
				
EV6	EV6F	CCW GAY CAY TTT GAY GGR TAY MAR C	4559–4583	sense
	EV6R	AHC CRT ACT GVV CVA CRT CHY C	5738–5759	antisense
				
EV7	EV7F	GTT GAG CGH CAC YTS AAY AGA GY	5216–5238	sense
	EV7R	GYA RRT CYA ACC CAT ACT TRT CC	6373–6395	antisense
				
EV8	EV8F	CAT YAA GAA RAR RGA CAT YYT BG	6310–6332	sense
	EV8R	TTG CTA TTC TGG TTA TAA CAA ATT TAC CC	7385–7413^#^	antisense

* The nucleotide position relative to the EV-71 MS87 strain

# Include 2 positions in poly-A tail

R = G/A      Y = T/C      M = A/C     K = G/T     S = G/C    W = A/T

H = not G    B = not A    V = not T    D = not C
